# *TERMINAL FLOWER1* is a breeding target for a novel everbearing trait and tailored flowering responses in cultivated strawberry (*Fragaria *× *ananassa* Duch.)

**DOI:** 10.1111/pbi.12545

**Published:** 2016-03-04

**Authors:** Elli Aurora Koskela, Anita Sønsteby, Henryk Flachowsky, Ola Mikal Heide, Magda‐Viola Hanke, Paula Elomaa, Timo Hytönen

**Affiliations:** ^1^ Department of Agricultural Sciences Viikki Plant Science Centre University of Helsinki Helsinki Finland; ^2^ Norwegian Institute of Bioeconomy Research Ås Norway; ^3^ Julius Kühn‐Institut Federal Research Centre for Cultivated Plants Institute for Breeding Research on Fruit Crops Dresden Germany; ^4^ Department of Ecology and Resource Management Norwegian University of Life Sciences Ås Norway; ^5^ Department of Biosciences Viikki Plant Science Centre University of Helsinki Helsinki Finland

**Keywords:** *Fragaria*, flowering, photoperiod, temperature, TERMINAL FLOWER1

## Abstract

The effects of daylength and temperature on flowering of the cultivated octoploid strawberry (*Fragaria *× *ananassa* Duch.) have been studied extensively at the physiological level, but information on the molecular pathways controlling flowering in the species is scarce. The flowering pathway has been studied at the molecular level in the diploid short‐day woodland strawberry (*F. vesca* L.), in which the *FLOWERING LOCUS T1* (*FvFT1*)–*SUPPRESSOR OF OVEREXPRESSION OF CONSTANS1* (*FvSOC1*)–*TERMINAL FLOWER1* (*FvTFL1*) pathway is essential for the correct timing of flowering. In this work, we show by transgenic approach that the silencing of the floral repressor *FaTFL1* in the octoploid short‐day cultivar ‘Elsanta’ is sufficient to induce perpetual flowering under long days without direct changes in vegetative reproduction. We also demonstrate that although the genes *FaFT1* and *FaSOC1* show similar expression patterns in different cultivars, the regulation of *FaTFL1* varies widely from cultivar to cultivar and is correlated with floral induction, indicating that the transcription of *FaTFL1* occurs at least partially independently of the *FaFT1*–*FaSOC1* module. Our results indicate that changing the expression patterns of *FaTFL1* through biotechnological or conventional breeding approaches could result in strawberries with specific flowering and runnering characteristics including new types of everbearing cultivars.

## Introduction

The cultivated strawberry (*Fragaria* × *ananassa* Duch.) is an agronomically important crop species grown under a wide range of environmental conditions. One aim in strawberry breeding programmes is to extend the cropping season. This can be achieved by breeding for early and late cultivars or everbearing cultivars, that is cultivars that flower perpetually throughout the growing season. Understanding the genetic mechanisms controlling flower induction in strawberry could help breeders in developing new cultivars with the desired flowering characteristics.

The physiology of flowering in cultivated strawberry (*Fragaria* × *ananassa* Duch.) has been extensively studied for nearly a century. Already the early work with the so‐called June‐bearing strawberries revealed that these are facultative short‐day (SD) plants, with floral induction taking place in SDs at temperatures above 15°C, behaving in day‐neutral manner at lower temperatures and flowering only poorly or not at all at high (>24°C) temperatures (Darrow and Waldo, 1934). However, different cultivars show variable environmental responses (Bradford *et al*., 2010; Heide, 1977), and some cultivars have an obligatory SD requirement for flower induction (Sønsteby and Heide, 2006). Environmental factors regulate also vegetative development; photoperiod and temperature control the fate of axillary meristems, which develop either into stolons (runners) or axillary leaf rosettes called branch crowns. Generally, branch crown development in SD cultivars is enhanced by environmental conditions favouring floral induction, and stolon formation is promoted by long days (LDs) and high temperature (Heide, 1977; Hytönen *et al*., 2004; Konsin *et al*., 2001; Mouhu *et al*., 2013).

Some strawberry cultivars flower perpetually and do not require SDs or low temperature for flowering. The flowering behaviour of these cultivars has been under debate, and this group has sometimes been nominated everbearers, day‐neutrals or LD plants, depending on the cultivars and experimental conditions used. Recently, it was convincingly shown that, in terms of flower induction, these strawberries are indeed obligatory LD plants at high temperature (27°C), quantitative LD plants at intermediate temperature and truly day‐neutrals only at cool (9°C) temperature (Sønsteby and Heide, 2007). As in SD strawberries, the flowering response in LD strawberries is dependent on the interaction of photoperiod and temperature in a cultivar‐specific manner, which may be the reason for confusion found in the literature. The vegetative responses in LD strawberries are more varying; stolon formation is promoted by high temperature and either by SDs or LDs, depending on the cultivar (Sønsteby and Heide, 2007).

Although flowering in the cultivated strawberry has been studied in detail at the physiological level, only few reports on the genetics of flowering in this species exist. Studies aimed at elucidating the genetic basis of the everbearing character via genetic mapping have reached varying results. The earliest reports suggested that the trait is controlled by a single dominant locus (Ahmadi *et al*., 1990; Sugimoto *et al*., 2005), while later it was proposed that the everbearing character is controlled by several QTLs (Weebadde *et al*., 2008). Two recent mapping studies by Gaston *et al*. (2013) and Castro *et al*. (2015) have identified a major QTL controlling both the everbearing and runnering traits, located on LGIVb‐f of the cultivated strawberry. Unfortunately, these two studies used different markers and mapping populations and it is therefore not possible to determine whether the QTLs are the same. Moreover, these studies did not suggest candidate genes for the everbearing trait, and there are no studies confirming the function of any flowering‐related gene in the cultivated strawberry.

As the genetics in the octoploid cultivated strawberry are notoriously complex, the closely related diploid wild strawberry *F. vesca* L. has been used in functional genetic studies for elucidating the molecular pathways controlling flowering in *Fragaria*. In *F. vesca*, both SD and LD genotypes exist and the flowering responses of these two genotypes are similar to those observed in the cultivated strawberry. SD genotypes initiate flowers photoperiod independently at low temperatures, are obligatory SD plants at temperatures between 13 and 20°C and are inhibited to flower at higher temperatures (Heide and Sønsteby, 2007; Rantanen *et al*., 2015). In contrast, flowering in the LD genotypes is promoted by LDs and temperature above 18°C, and delayed by SDs at cool (11°C) and high (27°C) temperatures (Mouhu *et al*., 2009; Sønsteby and Heide, 2008). The comparable physiological responses in *F. vesca* and the cultivated strawberry suggest that the principles of the molecular control of flowering in the two strawberry species are similar, and therefore, the use of the diploid *F. vesca* as a model species is plausible.

The genetics of the everbearing trait in *F. vesca* was studied already in the 1960s by Brown and Wareing (1965), who found that the everbearing character was caused by a recessive single gene termed *SEASONAL FLOWERING LOCUS* (*SFL*). More recently, it was shown by Koskela *et al*. (2012) that the recessive everbearing trait is caused by a lack‐of‐function mutation in the coding sequence of a *F. vesca* homologue of *TERMINAL FLOWER 1* (*FvTFL1*), a gene that represses flowering in a range of species, including Arabidopsis (Ohshima *et al*., 1997), apple (*Malus × domestica*; Kotoda *et al*., 2006; Flachowsky *et al*., 2012), roses (*Rosa* sp.; Iwata *et al*., 2012) and maize (*Zea mays;* Danilevskaya *et al*., 2010). It was further shown that the expression of *FvTFL1* is controlled by the photoperiodic pathway and is activated under LDs by *F. vesca SUPPRESSOR OF OVEREXPRESSION OF CONSTANS1* (*FvSOC1*; Mouhu *et al*., 2013). *FvSOC1* is in turn promoted by *Fragaria FLOWERING LOCUS T1* (*FvFT1*), which is expressed in leaf tissues exclusively under LDs (Koskela *et al*., 2012; Rantanen *et al*., 2014). Also an *F. vesca* homologue of *CONSTANS* (*FvCO*) has been identified, but its role in the photoperiodic regulation of *FvFT1* is unclear (Rantanen *et al*., 2014). The *FvFT1*–*FvSOC1*–*FvTFL1* pathway is activated under LDs in both SD and LD genotypes but leads to different flowering responses. In SD genotypes, LDs up‐regulate the expression of functional *FvTFL1*, which overrides the floral activator function of *FvFT1* and flowering is allowed only under SDs when *FvTFL1* is down‐regulated. In contrast, the absence of functional *FvTFL1* leads to *FvFT1*‐mediated floral induction in LD genotypes grown under LDs (Koskela *et al*., 2012; Rantanen *et al*., 2014). In both genotypes, the floral meristem identity genes *F. vesca APETALA1* (*FvAP1*) and *FRUITFUL1* (*FvFUL1*) are up‐regulated at the time of floral induction (Koskela *et al*., 2012; Mouhu *et al*., 2009, 2013).

The temperature‐controlled flowering pathway in the diploid strawberry has been studied recently by Rantanen *et al*. (2015), who showed that the effects of photoperiodic and temperature pathways converge at the regulation of *FvTFL1*. In *F. vesca*, cool temperature (<13°C) down‐regulates *FvTFL1* independently of photoperiod and SDs are required for floral induction only at intermediate temperatures (14–18°C). At high temperature (23°C), flowering is prevented because *FvTFL1* is up‐regulated independently of the photoperiodic pathway by an unknown activator.

In this work, we take advantage of the knowledge on molecular pathways regulating flowering in the diploid *F. vesca* and use functional and gene expression analysis to explore whether the pathways function similarly in the octoploid cultivated strawberry. We provide functional evidence of the role of *F.  × ananassa* homologue of *TFL1* as a floral repressor and show that *FaTFL1* regulation is associated with floral induction under a range of environmental conditions. Our results suggest that *FaFT1* and *FaSOC1* mRNA levels in the cultivated strawberry respond similarly to changes in environmental conditions as the photoperiodic pathway genes *FvFT1* and *FvSOC1* in the diploid *F. vesca*, but their expression does not always correlate with the *FvTFL1* mRNA levels.

## Results

### Silencing *FaTFL1* in ‘Elsanta’

It has been demonstrated in *F. vesca* that the silencing of *FvTFL1* eliminates the SD requirement for flowering (Koskela *et al*., 2012). To elucidate whether TFL1 is a floral repressor also in the cultivated strawberry, we transformed the short‐day cultivar ‘Elsanta’ using *TFL1*‐RNAi construct described by Koskela *et al*. (2012). The first transformation experiments resulted in several putative transgenic shoots originating from independent transformation events. Two shoots were positively tested for the presence of transgenic DNA sequences by PCR (Figure S1). Both shoots were clonally propagated to obtain the transgenic lines F138 and F139. These lines were found to contain at least one copy of the transferred T‐DNA by Southern hybridization (Figure S1).

**Figure 1:**
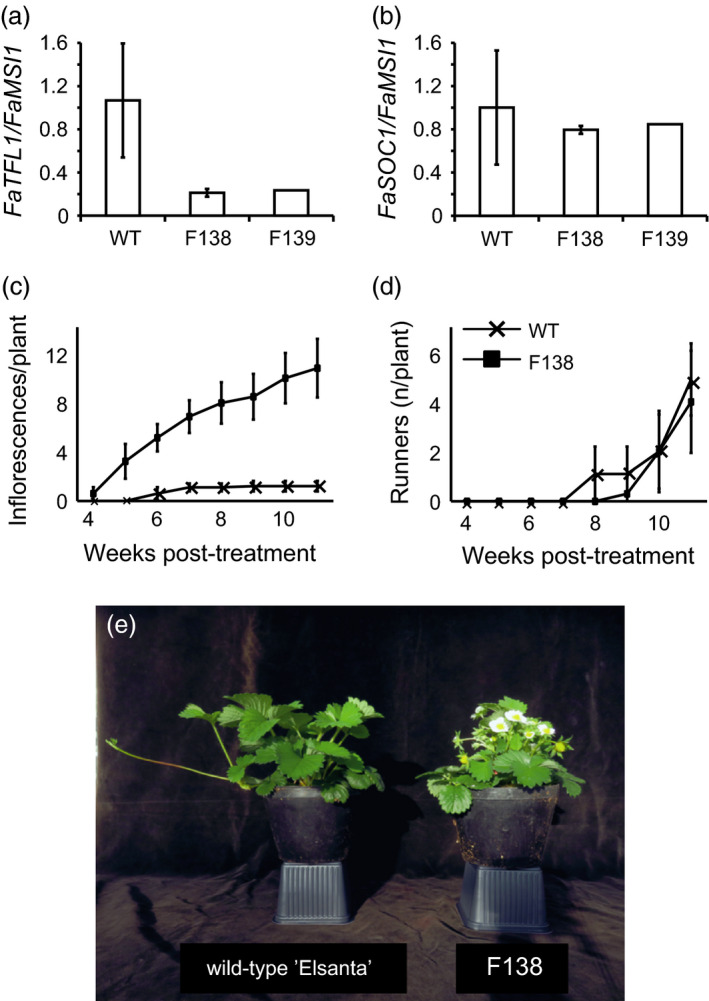
Phenotypic characterization of transgenic ‘Elsanta’ lines with silenced *FaTFL1*. (a–b) Relative expression of *FaTFL1* (a) and *FaSOC1* (b) in young runner apices of wild‐type ‘Elsanta’ and transgenic lines F138 and F139; (c–d) cumulative number of inflorescences (c) and runners (d) per plant after the flower induction and chilling treatments in wild‐type ‘Elsanta’ and the transgenic line F138; (e) representative plants of wild‐type ‘Elsanta’ and the transgenic line F138 subjected to artificial growth cycle. In (a) and (b), *n *= 3; in (c) and (d) *n *= 10, in (c–d) error bars indicate ± SD.

Silencing of *FaTFL1* in the two transgenic lines was further confirmed by quantitative RT‐PCR. To reliably compare the expression level of *FaTFL1* between the wild type and the transgenic plants, we needed to obtain vegetative apical tissues. As the transgenic plants flowered very early in LDs, we decided to use young runner apices without visible flower buds. In runner apices of LD‐grown F138 and F139, *FaTFL1* expression was strongly down‐regulated, whereas *FaTFL1* mRNA was expressed at a high level in wild‐type ‘Elsanta’ (Figure 1a). Expression of the floral integrator gene *FaSOC1* putatively upstream of *FaTFL1* on the photoperiodic flowering pathway was not significantly affected in the transgenic lines (Figure 1b).

The genome of *F. vesca*, a putative diploid progenitor species of *F*. * × ananassa*, harbours seven genes belonging to the same PEB (phosphatidyl ethanolamine‐binding) protein family as *FvTFL1* (Mimida *et al*., 2012). Therefore, it was of interest to confirm that the *TFL1*‐RNAi construct introduced into ‘Elsanta’ would silence only the transcripts orthologous to *TFL1*. We searched the Kazusa DNA Research Institute's database for *F*. * × ananassa* coding sequences and identified three coding sequences with similarity to the *TFL1*‐RNAi fragment. Phylogenetic analysis revealed that these sequences were the *F*. * × ananassa* homologues of *TFL1*,* CENTRORADIALIS1* (*CEN1*) and *CEN2* (Figure S2a). As *FaCEN2* showed nearly 80% identity with the *TFL1*‐RNAi fragment (Figure S2a), we decided to examine whether the *TFL1*‐RNAi construct would silence also *FaCEN2*. However, we were unable to detect notable changes in the expression level of *FaCEN2* between the wild‐type ‘Elsanta’ and the transgenic lines (Figure S2b).

Flowering phenotypes of the transgenic lines were assessed under LDs at 18°C. The transgenic line F138 started flowering 78.4 ± 5.9 days after moving the plants to the greenhouse, whereas ‘Elsanta’ remained vegetative in LDs. Some of the F139 plants started flowering already *in vitro* (Figure S3a). When the line F139 was grown under greenhouse conditions (LD, 18°C), flowering was observed after approximately 125 days. No floral buds were observed in the wild‐type ‘Elsanta’ plants after 150 days (Figure S3b).

As null mutation of *FvTFL1* causes continuous flowering in the woodland strawberry (Koskela *et al*., 2012), we tested the seasonality of flowering in ‘Elsanta’ *TFL1*‐RNAi line F138 by subjecting the plants to the artificial seasonal cycle in the greenhouse (see Experimental procedures). After SD and chilling periods, 60% of F138 plants flowered on 2nd of February, within 26 days of greenhouse forcing, whereas wild‐type ‘Elsanta’ started to flower 2 weeks later (Figure 1c and e). ‘Elsanta’ flowered only for a short period and all plants produced only 1 or 2 inflorescences. F138, however, continuously produced new inflorescences until the end of the experiment showing that the silencing of cultivated strawberry *TFL1* homologue causes continuous flowering in ‘Elsanta’. Both ‘Elsanta’ and F138 produced equal number of runners by the end of the experiment, although the first runners were observed 1 week earlier in ‘Elsanta’ than in F138 (Figure 1d).

### Photoperiodic responses in cultivars ‘Alaska Pioneer’, ‘Honeoye’ and ‘Polka’

To explore the photoperiodic regulation of key flowering time genes, three cultivars, ‘Honeoye’, ‘Polka’, and ‘Alaska Pioneer’, were selected based on their reportedly different flowering times. According to USDA National Plant Germplasm System, ‘Honeoye’ is considered an early mid‐season cultivar. Under Nordic conditions, ‘Honeoye’ is an early cultivar flowering approximately 1 week earlier than the mid‐season cultivar ‘Polka’ (Hytönen and Richterich, personal communication). ‘Alaska Pioneer’ was selected because it has been classified as an everbearing cultivar by USDA, but in our hands it requires SDs for flowering. Photoperiodic responses of these cultivars were studied in plants exposed to SDs (12 h of light) or LDs (18 h of light) at 18°C for 6 weeks. No flowering was observed in LDs, and in SDs, ‘Honeoye’ was the first to flower, followed by ‘Alaska Pioneer’ and ‘Polka’ (Table 1).

**Table 1:** Flowering of short‐day (SD) and long‐day (LD) grown plants of three strawberry cultivars. The plants were exposed to the specified daylengths at 18°C for 6 weeks and observed for flowering in LDs for the following 10 weeks. Days to anthesis was calculated from the end of daylength treatments. *N *= number of plants

Cultivar	Photoperiod (h)	*N*	Flowering plants (%)	Days to anthesis ± SD
Honeoye	SD 12	17	100	54.4 ± 3.5
	LD 18	10	0	>80
Alaska Pioneer	SD 12	12	100	65.2 ± 5.0
	LD 18	12	0	>80
Polka	SD 12	14	85.7	65.8 ± 6.8
	LD 18	10	0	>80

**Figure 2:**
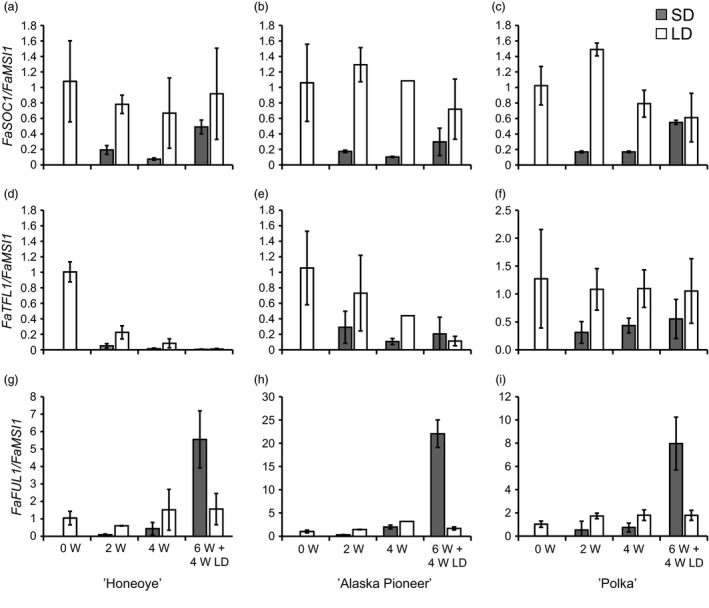
Expression of *FaSOC1*,* FaTFL1* and *FaFUL1* in the shoot apices of three strawberry cultivars. (a–c) Expression of *FaSOC1*; (d–f) expression of *FaTFL1*; (g–i) expression of *FaFUL1* in three strawberry cultivars grown in SDs or LDs. (a), (d) and (g) ‘Honeoye’; (b), (e) and (h) ‘Alaska Pioneer’; (c), (f), (i) ‘Polka’. Grey bars present SD (short day) and light bars LDs (long days). W = weeks under photoperiodic treatments; 6W + 4W LD = 6 weeks under photoperiodic treatments followed by 4 weeks under LDs. Error bars indicate ± SD (*n *= 3).

We examined diurnal expression of photoperiodically regulated genes *FaFT1* and *FaSOC1* in leaf tissues of the cultivar ‘Honeoye’. *FaFT1* was expressed exclusively in LDs, with two peaks, the first one 4 h after dawn and the second 20 h after dawn (Figure S4a). *FaSOC1* was expressed at a higher level in LDs than SDs (Figure S4b). Under SDs, there was no clear rhythm in *FaSOC1* expression, but in LDs the gene was slightly up‐regulated in the morning. In the shoot apex, on a longer time span, *FaSOC1* was down‐regulated after 2 weeks in SDs and remained low under short‐day conditions in all three cultivars (Figure 2a–c). When the plants were returned to LDs, expression levels of *FaSOC1* were restored to almost the same levels as in the LD‐treated plants.

*FaTFL1* expression showed more diverse patterns in the three cultivars analysed. In the beginning of the experiment, *FaTFL1* was expressed very strongly in the shoot apex tissues of ‘Honeoye’. In this cultivar, the floral repressor *FaTFL1* was gradually down‐regulated in both LDs and SDs, although down‐regulation occurred at a faster rate in SDs (Figure 2d). Similar trends were found also in ‘Alaska Pioneer’, although the reduction in the *FaTFL1* mRNA level under LD was weaker than in ‘Honeoye’ (Figure 2e). Only the cultivar ‘Polka’ showed a constant level of *FaTFL1* expression in LDs and the down‐regulation of the gene in SDs (Figure 2f). The floral meristem identity gene *FaFUL1* was up‐regulated only in the shoot apices of SD‐treated plants 4 weeks after the end of the SD treatment, concordant with the flowering observations (Figure 2g–i; Table 1).

### Interaction of photoperiod and temperature in ‘Elsanta’ and ‘Glima’

**Figure 3:**
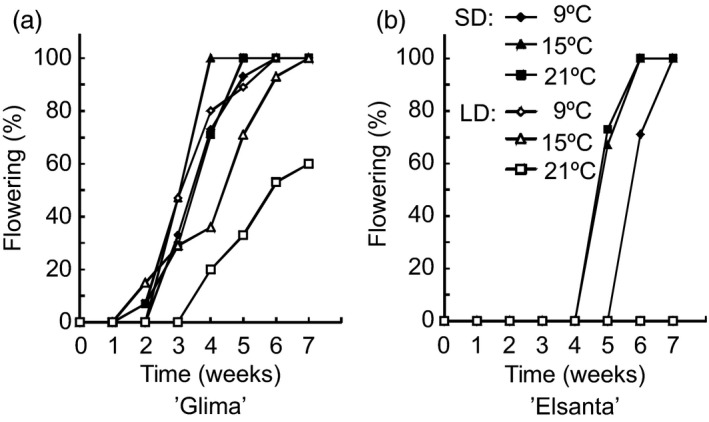
Percentage of flowering plants of ‘Glima’ and ‘Elsanta’. Clonally propagated plants of ‘Glima’ and ‘Elsanta’ were exposed to the specified conditions for 5 weeks, and flowering was then recorded under LD conditions at 20°C as days from the end of treatments. *n *= 12.

Next, we were interested in determining how the interaction of photoperiod and temperature affects flowering and the expression of flowering‐related genes in the cultivated strawberry. The cultivars ‘Elsanta’ and ‘Glima’ were selected for these treatments based on their different flowering responses; ‘Glima’ flowering readily in LD at temperatures below 21 °C (Heide, 1977), whereas ‘Elsanta’ is an obligatory SD plant and does not initiate flowers in LDs even at temperatures as low as 9 °C (Sønsteby and Heide, 2006). The experimental plants were subjected to SDs and LDs at three temperatures for 5 weeks. No flowering was observed in ‘Elsanta’ plants grown in LDs at any of the tested temperatures, while the SD‐treated plants grown at 15 °C and 21 °C flowered nearly simultaneously 34 days after the end of the SD treatment (Table 2; Figure 3a). Cool temperature (9 °C) delayed flowering by approximately 1 week. In ‘Glima’, the effect of photoperiod was much weaker than in ‘Elsanta’. ‘Glima’ plants grown in SDs at 9 °C and 21 °C flowered at the same time and produced roughly the same number of inflorescences, while 15 °C advanced flowering by approximately 4 days (Table 2; Figure 3b). LD treatment at 9 °C and 15 °C slightly delayed flowering as compared to the SD treatment at the same temperatures. The highest temperature of 21 °C was partially inhibitive to flowering under LDs in ‘Glima’, as only 60% of the plants flowered under these conditions and flowering was delayed by approximately 30 days. The effects of temperature, photoperiod and cultivar as well as their interactions were found to be statistically significant at α = 0.05 (Table 2).

**Table 2:** Flowering of ‘Elsanta’ and ‘Glima’ grown under short days (SDs) or long days (LDs) at different temperatures. The plants were treated under different photoperiods and temperatures for 35 days, after which they were moved to greenhouse (LD, 20°C) for flowering observations. Days to anthesis was calculated from the end of the treatments. *n *= 12

Cultivar	Photoperiod (h)	Temperature (°C)	Flowering plants (%)	Days to anthesis	No. of inflorescence/plant
Glima	SD 10	9	100	24.6	3.8
		15	100	20.8	4.9
		21	100	24.0	4.4
	LD 20	9	100	27.7	2.7
		15	100	28.9	2.2
		21	60	58.7	0.8
Elsanta	SD 10	9	100	41.8	1.0
		15	100	34.6	1.1
		21	100	34.1	1.2
	LD 20	9	0	>100	0
		15	0	>100	0
		21	0	>100	0
Probability level of significance (ANOVA) Source of variation					
Photoperiod (A)			0.003	0.004	0.006
Temperature (B)			<0.001	<0.001	n.s.
A × B			<0.001	<0.001	0.02
Cultivar (C)			<0.001	<0.001	<0.001
C × A			<0.001	<0.001	0.004
C × B			0.003	0.01	n.s.
A × B × C			0.04	0.03	n.s.

**Figure 4:**
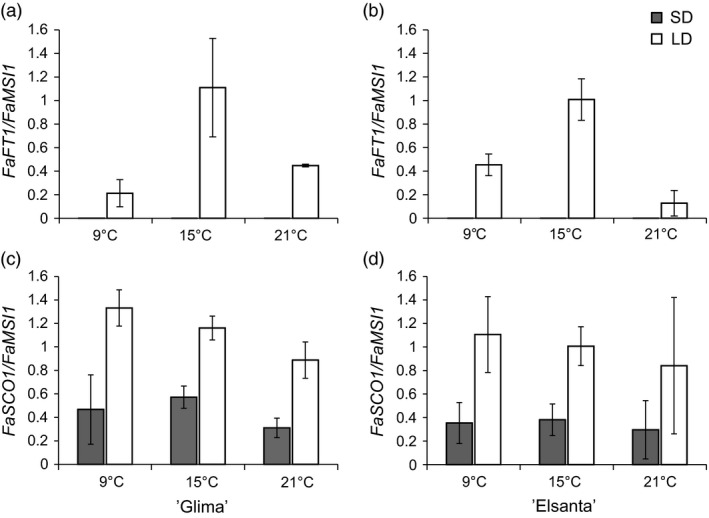
Expression of photoperiodically regulated genes in leaves of ‘Glima’ and ‘Elsanta’. (a–b) Expression of *FaFT1* in leaves of ‘Glima’ (a) and ‘Elsanta’ (b); (c–d) expression of *FaSOC1* in leaves of ‘Glima’ (c) and ‘Elsanta’ (d) grown under different daylength and temperature conditions for 30 days. Error bars indicate ± SD (*n *= 3).

To understand the effects of daylength and temperature on the regulation of flowering‐related genes, leaf and shoot apex samples of ‘Glima’ and ‘Elsanta’ were collected after 30 days under experimental conditions. In leaf tissues, *FaFT1* expression was strongly down‐regulated by SDs, being nearly undetectable in the SD samples (Figure 4a–b). However, temperature had an effect on *FaFT1* expression in both cultivars under LDs, the expression being highest at 15 °C. *FaSOC1* expression in leaf tissue was down‐regulated by SDs, and no obvious differences between cultivars and temperature treatments were observed (Figure 4c–d).

**Figure 5:**
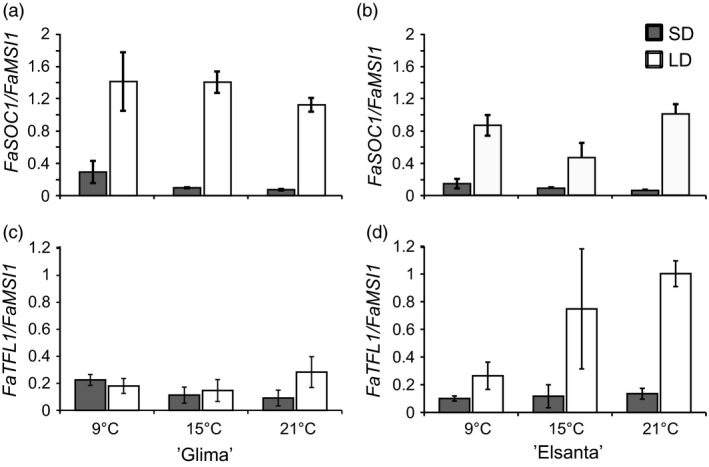
Expression of photoperiodically regulated genes in the shoot apices of ‘Glima’ and ‘Elsanta’. (a–b) Expression of *FaSOC1* in ‘Glima’ (a) and ‘Elsanta’ (b); (c–d) expression of *FaTFL1* in ‘Glima’ (c) and ‘Elsanta’ (d) grown under different daylengths and temperatures. Error bars indicate ± SD (*n *= 3).

In the shoot apices of both cultivars, *FaSOC1* expression was strongly down‐regulated by SDs, similarly as in the leaf samples (Figure 5a–b). Temperature did not affect *FaSOC1* expression levels in the apices of ‘Glima’, whereas in ‘Elsanta’ *FaSOC1* mRNA was slightly less abundant in the LD/15 °C samples than in the other LD samples. In addition to using our own primers for quantitative RT‐PCR, we measured *FaSOC1* relative expression with the primers described by Nakano *et al*. (2015). The expression patterns with the two primer pairs were nearly identical (Figure S5a–b).

The expression levels of *FaTFL1* were generally concordant with the flowering data. In ‘Glima’ (Figure 5c), expression of *FaTFL1* was lower than in ‘Elsanta’ (Figure 5d) in all treatments. Daylength had an effect on *FaTFL1* expression only at 21 °C, where LDs up‐regulated *FaTFL1* in correlation with delayed flowering (Figure 5, Table 2). In ‘Elsanta’, SDs down‐regulated *FaTFL1* at all tested temperatures, concordant with flowering phenotypes. However, *FaTFL1* transcript level in ‘Elsanta’ was quite low also in LDs at 9°C, although no flowering was observed in this treatment.

## Discussion

In this report, we show how the silencing of a key floral repressor *FaTFL1* leads to daylength‐independent flowering in the cultivated strawberry. We provide evidence that the expression of *FaFT1* and *FaSOC1* in the different cultivars of the cultivated strawberry responds similarly to environmental conditions as the *FvFT1* and *FvSOC1* genes in the diploid *F. vesca*, but the regulation of *FaTFL1* does not always coincide with the changes in the *FaFT1* and *FaSOC1* mRNA levels. We show that the regulation of *FaTFL1* expression in the cultivated strawberry cultivars is diverse and is associated with the varying flowering responses of the cultivars.

### *TFL1* is a floral repressor in the cultivated strawberry

Homologues of *TFL1* have been shown to act as repressors of flowering in a wide range of species including Arabidopsis (Bradley *et al*., 1997; Hanano and Goto, 2011), *Malus × domestica* (Flachowsky *et al*., 2012; Kotoda *et al*., 2006), *Arabis alpina* (Wang *et al*., 2011) and *F. vesca* (Koskela *et al*., 2012). In Arabidopsis, mutations at *TFL1* cause early flowering and conversion of the inflorescence meristem into floral meristem, leading to formation of the terminal flower (Bradley *et al*., 1997). Similarly, in *Arabis alpina*, silencing *AaTFL1* leads to formation of terminal flowers and also reduces the juvenile phase (Wang *et al*., 2011). Moreover, disruption of *TFL1* expression in apple reduces the duration of both the juvenile and vegetative phases and results in very early flowering (Kotoda *et al*., 2006). The gene is also reported to have a role in the control of yearly growth cycles in perennial plants such as Lombardy poplar (*Populus nigra* var. *italica*; Igasaki *et al*., 2008), roses (Iwata *et al*., 2012) and *F. vesca* (Koskela *et al*., 2012). In *F. vesca*,* FvTFL1* is photoperiodically regulated by the LD‐activated *FvFT1*–*FvSOC1* flowering pathway at temperatures between 13 and 18°C, leading to the repression of flowering in LDs (Mouhu *et al*., 2013; Rantanen *et al*., 2015). At cool temperatures, *FvTFL1* is repressed daylength independently and flowering is induced in both SDs and LDs, whereas high temperatures promote *FvTFL1* expression and inhibit flowering (Rantanen *et al*., 2015). To investigate the role of *FaTFL1* in repression of flowering in the cultivated strawberry, we analysed transgenic ‘Elsanta’ plants with silenced *TFL1*.

In *F. vesca*, silencing of *FvTFL1* leads to daylength‐independent flowering but is not involved in the photoperiodic control of vegetative development, that is development of runners (Koskela *et al*., 2012). Similarly, in the transgenic ‘Elsanta’ lines with *TFL1*‐RNAi construct, silencing of *TFL1* abolished seasonal regulation of growth cycles causing daylength‐independent perpetual flowering (Figure 1c and e), but did not affect the formation of runners in the transgenic lines (Figure 1d). These data provide strong evidence that *FaTFL1* functions as a floral repressor, which has no direct effect on the vegetative reproduction in the cultivated strawberry. The absence of connection between *TFL1* and vegetative vigour seems to be strawberry specific; in many other Rosaceous crop plants, including pear (Freiman *et al*., 2012) and apple (Flachowsky *et al*., 2012; Kotoda *et al*., 2006), reducing *TFL1* expression results in plants with greatly reduced vegetative growth.

Results from transgenic experiments in *F. vesca* indicated that FvSOC1 separately promotes runner formation through the activation of the gibberellin pathway and represses flowering by activating *FvTFL1* expression. However, FvTFL1 has no effect on the expression of *FvSOC1* or runner formation (Koskela *et al*., 2012; Mouhu *et al*., 2013). Similarly, the silencing of *FaTFL1* in ‘Elsanta’ did not change *FaSOC1* expression. Further studies are needed to reveal whether FaSOC1 also functions as a branching point in the genetic pathway controlling vegetative and generative reproduction in the cultivated strawberry. Based on our findings, flowering time in the cultivated strawberry could be extended by reducing *FaTFL1* expression either by means of conventional breeding, through transgenic procedures or genome editing (Xiong *et al*., 2015) without direct consequences in vegetative reproduction. Poor runner production in current everbearing cultivars (Sønsteby and Heide, 2007), which was reported to be caused by the same major QTL as the perpetual flowering habit itself (Gaston *et al*., 2013), is limiting their vegetative propagation in nurseries. Based on our data, the production of novel everbearing cultivars based on *FaTFL1* silencing could possibly solve this problem.

### Altered patterns of *FaTFL1* expression may contribute to the different flowering responses in the cultivated strawberry

*TFL1* down‐regulation has been shown to correlate with the expression of floral meristem identity genes and subsequent flowering in several perennial Rosaceous species, namely the diploid strawberry *F. vesca* (Koskela *et al*., 2012), *M. domestica* (Hättasch *et al*., 2008) and *Rosa* sp. (Iwata *et al*., 2012). Although the gene appears to have an important role in the control of flowering, the variation in its expression patterns within a certain species has not been studied. In this work, we analysed the variation in *FaTFL1* expression patterns in five octoploid strawberry cultivars. The results show striking cultivar‐dependent differences in the regulation of *FaTFL1* expression by environmental conditions.

*FaTFL1* down‐regulation correlated with flowering in cultivars ‘Polka’, ‘Glima’ and ‘Elsanta’. By contrast, down‐regulation of *FaTFL1* was detected under both daylengths in the early cultivar ‘Honeoye’ and in ‘Alaska Pioneer’ that flowered eleven days later, although the gene was expressed at a higher level in LDs than SDs at all time points (Figure 2). It appears contradictory that *FaTFL1* was down‐regulated in both SDs and LDs in these cultivars, although no flowering in LDs was observed (Table 1), and *FaFUL1* was up‐regulated only in SDs (Figure 2g–h). However, earlier experiments with ‘Honeoye’ have shown that the cultivar can be induced to flower daylength independently at 17°C, with LDs delaying flowering (Bradford *et al*., 2010). It is therefore possible that flowering could have eventually occurred in ‘Honeoye’ and ‘Alaska Pioneer’ also in LDs had the plants been observed for a longer time. Moreover, a recent study by Nakano *et al*. (2015) demonstrated similar daylength‐independent down‐regulation of *FaTFL1* in the Japanese octoploid strawberry cultivar ‘Nyoho’. The authors suggested that the observed down‐regulation of *FaTFL1* in ‘Nyoho’ could be due to high initial levels of *FaTFL1* caused by the conditions where the experimental plants were prepared, that is under high natural temperature. It seems likely that the initial level of *FaTFL1* mRNA was high in the young ‘Honeoye’ and ‘Alaska Pioneer’ plants used in the experiment, but this cannot be caused by high temperatures or high light conditions as the plants were prepared during winter under controlled greenhouse conditions. A more likely explanation, which should be studied further, is that the level of *FaTFL1* transcript is extremely high in young plants of these cultivars and is reduced in an age‐dependent manner. Age‐dependent reduction in *TFL1* mRNA has been earlier described in the perennial *A. alpina* (Wang *et al*., 2011), in which TFL1 expressed in the shoot apical meristem blocks flowering of young plants exposed to vernalization.

In *F. vesca, FvTFL1* is down‐regulated and flowering induced independently of the photoperiod below a critical limit of 13°C, whereas high temperature (23°C) represses flowering by up‐regulating *FvTFL1* (Rantanen *et al*., 2015). Previous experiments with the cultivated strawberry cultivars have suggested that temperature may control flowering through a similar mechanism, although critical temperature limits differ widely depending on the cultivar. Sønsteby and Heide (2006) have shown that even temperature as low as 9°C is not sufficiently low for induction of flowering in ‘Elsanta’, and temperature of 27°C is required for repressing flowering in SDs. In contrast, ‘Glima’ induces flowers independent of daylength at 18°C, and temperature of 24°C is not high enough for complete floral repression (Heide, 1977). Our results are in line with the earlier physiological studies, as SDs down‐regulated *FaTFL1* and induced flowering in ‘Elsanta’ at all tested temperatures (the highest being 21°C), whereas in ‘Glima’, *FaTFL1* mRNA levels were lower in LDs than in ‘Elsanta’ and flowering occurred in all photoperiod–temperature combinations (Table 2 and Figure 5). However, LD caused a partial floral repression at the highest temperature, which was associated with increased *FaTFL1* mRNA level compared to SD. Contradictory to the observations in *F. vesca*, cool temperature of 9°C did not induce flowering in ‘Elsanta’ although *FaTFL1* was down‐regulated. It is possible that although the level of *FaTFL1* in ‘Elsanta’ under the LD/9°C treatment was low, it was still sufficient to repress flowering in this cultivar. Several studies in *Arabidopsis* have shown that TFL1 is a stronger repressor of flowering under cool temperature (Hanano and Goto, 2011; Kim *et al*., 2013; Strasser *et al*., 2009), and the same could be true in the cultivated strawberry. Another possibility is that the *FaTFL1* homoeologs not detected by our RT‐qPCR primers were expressed at a higher level. Future research with subgenome specific primers should be carried out to show whether the homoeologs are expressed at different levels.

### Is the *FaFT1*–*FaSOC1–FaTFL1* pathway present in the cultivated strawberry?

The long‐day activated photoperiodic flowering pathway has been well characterized in *F. vesca*. In *F. vesca*, LDs activate *FvFT1* expression in the leaf leading to *FvSOC1* activation in the shoot apex. By contrast, SDs strongly down‐regulate *FvFT1* expression in the leaf and cause a gradual down‐regulation of *FvSOC1* in the shoot apex leading to the reduction in *FvTFL1* mRNA levels and flower induction (Koskela *et al*., 2012; Mouhu *et al*., 2013).

To elucidate whether the LD‐activated *FT1*–*SOC1* pathway shows similar expression patterns in the cultivated strawberry, we studied the expression of these genes in several strawberry cultivars. In all tested cultivars, *FaFT1* was expressed in leaf tissues exclusively in LDs (Figures 4 and S4). Moreover, the diurnal rhythm of *FaFT1* in ‘Honeoye’ showed a similar pattern to the rhythmical expression of *FvFT1* observed in *F. vesca* (Koskela *et al*., 2012). Interestingly, *FaFT1* expression in the cultivated strawberry is regulated also by temperature, the expression being highest at intermediate temperature (Figure 4). Similar results were reached in *F. vesca* by Rantanen *et al*. (2015), who showed that *FvFT1* is regulated by ambient temperature, although no correlation between the flowering response and *FvFT1* expression was observed between the temperatures. Our results on *FaFT1* regulation in cultivated strawberry support the recent reports by Nakano *et al*. (2015) and Nakajima *et al*. (2014), who reported higher *FaFT1* expression in LDs than SDs in the Japanese cultivars ‘Nyoho’ and ‘Tochiotome’, respectively. However, Nakano *et al*. (2015) did not detect clear daylength‐dependent regulation of *FaSOC1*, in contrast with our finding that *FaSOC1* is down‐regulated in SDs in both leaves and apical tissues (Figures 2, 4 and 5). We first hypothesized that the discrepancy could be caused by different primers used for quantitative RT‐PCR. When we tested the *FaSOC1* primers of Nakano *et al*. (2015), the expression patterns were nearly identical to those obtained with our own primers (Figures 5 and S5). It therefore appears that the observed lack of photoperiodic regulation of *FaSOC1* in the Japanese cultivar ‘Nyoho’ reflects a true difference in *FaSOC1* regulation as compared to European strawberry cultivars.

In general, we detected lower *FaTFL1* expression levels in SD‐ than in LD‐grown shoot apices except in ‘Glima’ indicating that, similarly to *F. vesca* (Mouhu *et al*., 2013), the photoperiodic control of *FaTFL1* is present in the cultivated strawberry. In many cases, however, the changes in the *FaTFL1* mRNA levels did not clearly reflect the changes in the expression of *FaFT1* and *FaSOC1* indicating that other mechanisms are involved in the regulation of *FaTFL1*. This is clearly true also in *F. vesca*, in which strong coincidence in the photoperiodic control of *FvSOC1* and *FvTFL1* only occurs in quite narrow temperature range (Rantanen *et al*., 2015). Our results suggest that the identification of the genetic variation causing variable *TFL1* expression patterns in strawberries may open new possibilities for tailoring flowering responses.

## Conclusions

The results presented here suggest that *FaTFL1* acts as a floral repressor in the cultivated strawberry, and its down‐regulation is correlated with subsequent floral induction in most cases. Cultivars exhibit large differences in the regulation of *FaTFL1*; some cultivars may down‐regulate *FaTFL1* in an age‐dependent manner, whereas in other cultivars *FaTFL1* down‐regulation is more dependent on environmental conditions. As the diverse expression patterns do not seem to arise from differential activity of the photoperiodically controlled *FaFT1*–*FaSOC1* pathway, the elucidation of other upstream regulators of *FaTFL1* or functional allelic variation in the *FaTFL1* locus could provide valuable information for targeted plant breeding. Decreasing *FaTFL1* expression levels either via conventional breeding or using transgenic approaches could result in novel everbearing or earlier flowering cultivars without direct effect on vegetative reproduction through runners.

## Experimental procedures

### Plant transformation

For plant transformation, axillary shoot cultures of the octoploid strawberry (*F*. × *ananassa* Duch.) cultivar ‘Elsanta’ were used. The plant material was propagated *in vitro* on shoot proliferation medium containing MS salts and vitamins (Murashige and Skoog, 1962) supplemented with 0.1 mg/L 6‐benzylaminopurine (BAP), 0.01 mg/L indole‐3‐acetic acid (IAA), 30 g/L sucrose and 0.45% Difco Bacto‐agar (Difco, Heidelberg, Germany). Plants were grown in a culture chamber (16 h light at 21°C and 8 h dark at 16°C) and subcultured every 3–4 weeks. Plant transformation was performed using the *Agrobacterium tumefaciens* strain EHA105 complemented with the binary plasmid vector pK7GWIWG2D(II)_TFL1‐RNAi (Koskela *et al*., 2012) as described by Fischer *et al*. (2014). Regenerated meristems were excised 9–12 weeks postinoculation. Regenerated shoots were propagated and subcultured on shoot proliferation medium with 500 mg/L timetin and 300 mg/L kanamycin at 16 h of light at 21°C and 8 h of darkness at 16°C. Rooted plantlets (*n *= 5 for each transgenic line) were grown in 5 cm plastic pots in the greenhouse in LD conditions at 18°C.

### PCR‐based detection of transgenic sequences

Genomic DNA was extracted from *in vitro* leaves of the transgenic lines and wild‐type ‘Elsanta’ using the DNeasy Plant Mini Kit (Qiagen, Hilden, Germany). PCR was performed in 25 μL volume containing 10 ng DNA, 1 × DreamTaq^™^ buffer, 0.2 mm deoxynucleoside triphosphates (dNTPs), 0.5 μm of each primer and 0.5 U DreamTaq^™^ DNA polymerase (MBI Fermentas, St. Leon‐Roth, Germany). The PCR started by initial denaturation at 94°C for 4 min, followed by 33 cycles of 30 s of denaturation at 94°C, 1 min of annealing at 56°C and 1.5 min of extension at 72°C, and a final extension at 72°C for 7 min. PCR was performed using a MyCycler^™^ thermocycler (Bio‐Rad, Hercules, CA). Primers used for detecting the *nptII* marker gene and the chimeric *TFL1* hairpin construct are listed in Table S1.

### Plant materials, experimental conditions and sampling

Plants for all experiments were propagated clonally from runner plants of LD‐grown mother plants. In the artificial seasonal cycle experiment with wild‐type ‘Elsanta’ and *TFL1*‐RNAi line F138, the clonally propagated plants (*n *= 10) were grown for 1 month in LDs in the greenhouse, after which they were subjected to natural SDs in an unheated greenhouse in Helsinki from 25 September 2014 to 13 November 2014. Then, greenhouse temperature was set to 6°C for the following 56 days (1344 chilling hours) to release dormancy. After chilling, plants were forced in the greenhouse under 18‐h photoperiod. Temperature was first set to 12°C and then gradually increased to 18°C during the first 2 weeks. Inflorescences with open flowers and runners were counted and removed weekly.

For the environmental treatments, runner‐propagated plants of ‘Alaska Pioneer’, ‘Honeoye’ and ‘Polka’ were first grown in LDs (18 h light, 18°C) for 3 weeks. Plants were then exposed to either SD (12 h, 18°C) or LD (18 h, 18°C) conditions for 6 weeks and then moved to LD at 18°C for flowering observations. For analysing temporal changes in gene expression, three biological replicates of apex samples were collected at ZT9 0, 2, 4 and 10 weeks after the beginning of the daylength experiments, each sample containing the main apex of three individual plants. Leaf samples for analysing circadian rhythmic expression in cultivar ‘Honeoye’ were collected 3 weeks after the beginning of daylength treatments at 4, 8, 12, 16, 20, 24 and 28 h after the ‘first’ subjective dawn. Leaf samples consisted of the middle leaflet of a fully opened leaf, and three biological replicates were collected.

Clonally propagated plants of cultivars ‘Glima’ and ‘Elsanta’ were rooted directly into 10 cm pots and grown in a greenhouse in LDs (20 h) at 20°C for 4 weeks. The plants were exposed to combinations of temperature (9°C, 15°C and 21°C) and daylength (SD = 10 h, and LD = 20 h) for 5 weeks, after which the plants were moved to LD at 20°C for flowering observations. Using low‐intensity (7 μmol quanta/m^2^/s) incandescent light for daylength extension, the daily integral differed only by 0.5% between the daylength treatments. Leaf and apex samples for RNA extractions were collected at noon (+2 h) on day 30 with three biological replicates. Each leaf replicate was pooled from the youngest fully open leaves of 4 plants, and each apex sample contained the main shoot apices of four plants. All samples were immediately frozen in liquid nitrogen and stored at −80°C.

### RNA extraction and quantitative RT‐PCR

Apex samples were milled in Retsch MM400 ball mill (Retsch GmbH, Düsseldorf, Germany) at 28/second for 30 s. Leaf samples were ground in liquid nitrogen using a mortar and a pestle. RNA was extracted using the CTAB‐extraction buffer based pine tree method (Monte and Somerville, 2002). cDNA was synthesized from 1 μg total‐RNA using Superscript III reverse transcriptase (Invitrogen, Thermo Fisher Scientific, MA). RT‐PCRs were performed with three technical replicates and three biological replicates with SYBR Green I master mix (Roche Deutschland Holding GmbH, Mannheim, Germany) run in Lightcycler 480 instrument (Roche). Quantitative RT‐PCR was run in a total volume of 10 μL with final concentration of 1 × SYBR Green Master Mix I (Roche) and 0.45 μm primers. The RT‐qPCR profile is described in Figure S6. A total of 3.5 μL of diluted cDNA (final volume 150 μL) was used in each reaction. Relative expression levels were calculated by ΔΔCt method (Pfaffl, 2001) with *FaMSI1* (*Fragaria × ananassa* homologue of *MULTICOPY SUPPRESSOR OF IRA1*) as normalization gene. Primers used for RT‐qPCR are listed in Table S1.

## Supporting information


**Table S1** Primer sequences used in the experiments.
**Figure S1** Evaluation of transgenic strawberry clones for the presence of the transferred DNA sequences.
**Figure S2** Specificity of silencing *FaTFL1* using the *FvTFL1*‐RNAi construct.
**Figure S3** Flowering phenotype of the transgenic line F139.
**Figure S4** Circadian rhythms of *FaFT1* and *FaSOC1*.
**Figure S5** *FaSOC1* expression in ‘Glima’ and ‘Elsanta’ shoot apices.
**Figure S6** RT‐qPCR programme used for analyzing gene expression.
