# Supplementary material for: TERMINAL FLOWER1 is a breeding target for a novel everbearing trait and tailored flowering responses in cultivated strawberry (Fragaria × ananassa Duch.)
Source: Plant Biotechnol J. 2016 Mar 4;14(9):1852–61. doi: 10.1111/pbi.12545 (PMC5069601; doi:10.1111/pbi.12545)
Supplement: Supplementary file 1 — Table S1 Primer sequences used in the experiments. Figure S1 Evaluation of transgenic strawberry clones for the presence of the transferred DNA sequences. Figure S2 Specificity of silencing FaTFL1 using the FvTFL1‐RNAi construct. Figure S3 Flowering phenotype of the transgenic line F139. Figure S4 Circadian rhythms of FaFT1 and FaSOC1. Figure S5 FaSOC1 expression in ‘Glima’ and ‘Elsanta’ shoot apices. Figure S6 RT‐qPCR programme used for analyzing gene expression. [file PBI-14-1852-s001.docx]

# *TERMINAL FLOWER1* is a breeding target for a novel everbearing trait and tailored flowering responses in cultivated strawberry (*Fragaria* × *ananassa* Duch.)

Elli Aurora Koskela, Anita Sønsteby, Henryk Flachowsky, Ola Mikal Heide, Magda-Viola Hanke, Paula Elomaa, Timo Hytönen

**Supplementary data**

***Supplementary Methods***

### Southern hybridization

Southern hybridization experiments were performed using 5 µg of DNA digested with 100 U *Hind*III (MBI Fermentas) at 37°C overnight. The restricted DNA was separated on a 0.8% agarose gel and blotted onto a positively charged nylon membrane (Roche Deutschland Holding GmbH, Mannheim, Germany). The membrane was hybridized with a PCR-amplified, digoxygenin-labeled probe of *nptII* generated using the primers nptII_F/R and the PCR DIG Probe Synthesis Kit (Roche Deutschland Holding GmbH). Detection was performed using Anti-DIG-AP (Roche Deutschland Holding GmbH) and ECF^TM^ substrate (Amersham Biosciences Europe GmbH, Freiburg, Germany) on a ChemiDoc^TM^ XRS System (Bio-Rad Laboratories GmbH).

***Supplementary Tables***

**Supplementary Table 1**. Primer sequences used in the experiments.

| Primer name | Forward primer 5’-3’ | Reverse primer 5’-3’ | Primer pair efficiency |
| --- | --- | --- | --- |
| nptIIF | ACAAGATGGATTGCACGCAGG | AACTCGTCAAGAAGGCGATAG | n/a |
| TFL1hairpin | AAAAAGCAGGCTTGTTTGGCCTTGGCATCTGC | AGAAAGCTGGGTTCTGCAGTCACCGCCAAACC | n/a |
| RT–MSI1 | TCCCCACACCTTTGATTGCCA | ACACCATCAGTCTCCTGCCAAG | 1,95 |
| RT-TFL | CTGGCACCACAGATGCTACA | AACGGCAGCAACAGGAAC | 1,95 |
| RT-SOC1 | ACTTGCTGGGTTCATTTTCC | GAGCTTTCCTCTGGGAGAGA | 1,98 |
| RT- FUL1 | GCAGTGCATGAATCCCTTTC | GCTGGTGATTTTGGAGCTTG | 2,09 |
| RT-FT1 | CAATCTCTTGGCCGAAAACT | TGAGCTCAAACCTTCCCAAG | 1,94 |
| TFL1 gene-specific | AAAAAGCAGGCTCTGTACAACCTTTTCTCTTCTCCCTC | AGAAAGCTGGGTCCTCCCTGCAAGGTGCCTA | n/a |

## *Supplementary Figures*

**
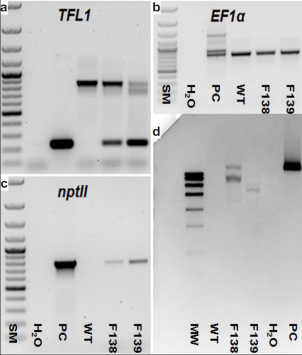
 Supplementary Figure 1.** **Evaluation of transgenic strawberry clones for the presence of the transferred DNA sequences.** The presence of transgenic DNA sequences was tested by PCR for the chimeric *TFL1* hairpin gene construct (a) and for *nptII* (c). The quality of the DNA was tested using primers specific for the housekeeping gene EF1α (b). Integration of transgenic DNA sequences into the strawberry genome was tested by Southern hybridization using an *nptII* specific probe (d). SM, size marker (GeneRuler^TM^ 100 bp Plus; Thermo Scientific, Schwerte, Germany); H_2_O, blank control using ddH_2_O as template; PC, positive control using plasmid DNA containing the expected PCR fragment; WT, wild type DNA of 'Elsanta' used as negative control; F138, DNA of transgenic clone F138; F139, DNA of transgenic clone F139; MW, DNA Molecular Weight Marker VII (Roche Deutschland Holding GmbH, Mannheim, Germany).

**
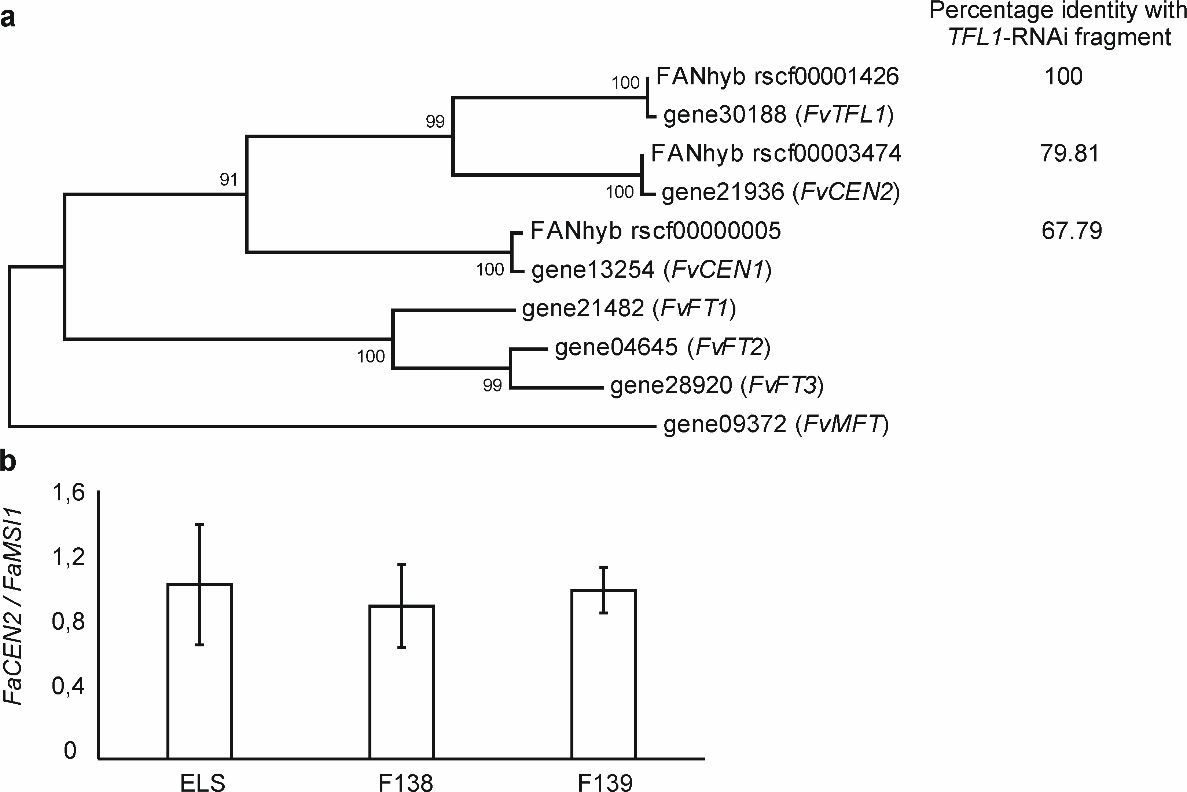
**

**Supplementary Figure 2. Specificity of silencing *FaTFL1* using the *FvTFL1*-RNAi construct.** a) Identification of *F*. × *ananassa* coding sequences similar to the *TFL1*-RNAi construct; FANhyb sequences are BLAST hits from the Kazusa DNA Research Institute’s database against the *TFL1*-RNAi fragment; *F. vesca* sequences were retrieved from the Genome Database for Rosaceae. The phylogenetic tree was constructed with 1000 bootstrap replicates using the maximum likelihood method of MEGA6 software (Tamura *et al*., 2013). b) Relative expression of *FaCEN2* in young runner apices of ‘Elsanta’ (ELS) and transgenic lines F138 and F139; n = 3, error bars indicate ± standard deviation.

**
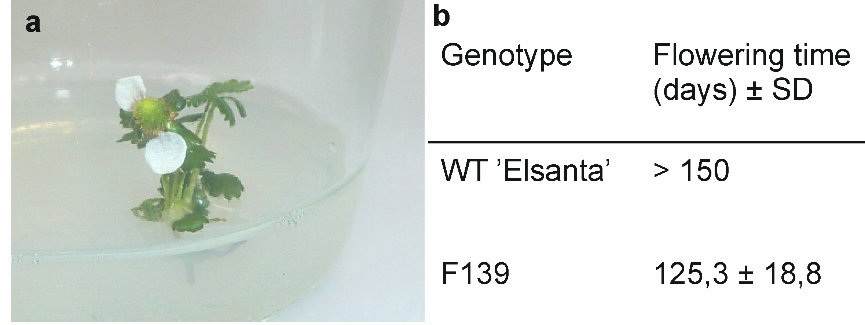
**

**Supplementary Figure 3. Flowering phenotype of the transgenic line F139.** (a) *In vitro* flowering in the transgenic line F139. (b) Flowering time of wild type 'Elsanta' and line F139 grown under LDs (n = 6).

**
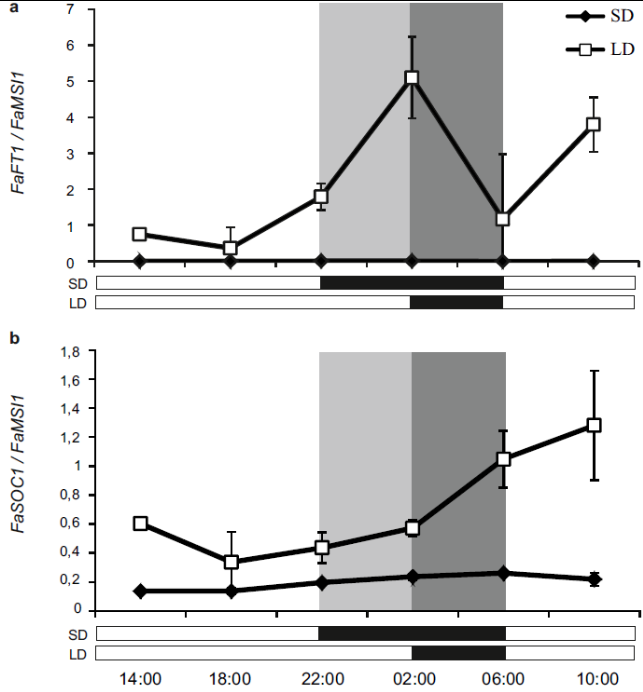
**
**Supplementary Figure 4**. **Circadian rhythms of *FaFT1* and *FaSOC1***. Circadian rhythms of *FaFT1* (a) and *FaSOC1* (b) in leaves of 'Honeoye' grown under SDs (short days) or LDs (long days) at 18°C. Samples were collected after three weeks under the photoperiodic treatments, error bars indicate ± standard deviation (n = 3).

**

**

**Supplementary Figure 5. *FaSOC1* expression in ‘Glima’ and ‘Elsanta’ shoot apices.** Expression of *FaSOC1* in the shoot apices of ‘Glima’ (a) and ‘Elsanta’ (b) determined by the primers described in Nakano et al. (2015). Error bars indicate ± standard deviation (n = 3).

**Supplementary Figure 6.** RT-qPCR programme used for analyzing gene expression.
